# Testing telediagnostic thyroid ultrasound in Peru: a new horizon in expanding access to imaging in rural and underserved areas

**DOI:** 10.1007/s40618-021-01584-7

**Published:** 2021-05-10

**Authors:** T. J. Marini, S. L. Weiss, A. Gupta, Y. T. Zhao, T. M. Baran, B. Garra, I. Shafiq, D. C. Oppenheimer, M. S. Egoavil, R. L. Ortega, R. A. Quinn, J. Kan, A. M. Dozier, L. Tamayo, C. Carlotto, B. Castaneda

**Affiliations:** 1 University of Rochester Medical Center, 601 Elmwood Ave, Box 648, Rochester, NY 14642 USA; 2 Medical Imaging Ministries of the Americas, 10810 Lake Minneola Shores, Clermont, FL 34711 USA; 3 Medical Innovation and Technology, Calle Los Libertadores 635, 15046 San Isidro, Peru; 4 Pontifica Universidad Catolica del Peru, Av. Universitaria 1801, 15088 San Miguel, Peru

**Keywords:** Global health, Goiter, Telemedicine, Thyroid cancer, Thyroid ultrasound, Ultrasound

## Abstract

**Purpose:**

Thyroid ultrasound is a key tool in the evaluation of the thyroid, but billions of people around the world lack access to ultrasound imaging. In this study, we tested an asynchronous telediagnostic ultrasound system operated by individuals without prior ultrasound training which may be used to effectively evaluate the thyroid and improve access to imaging worldwide.

**Methods:**

The telediagnostic system in this study utilizes volume sweep imaging (VSI), an imaging technique in which the operator scans the target region with simple sweeps of the ultrasound probe based on external body landmarks. Sweeps are recorded and saved as video clips for later interpretation by an expert. Two operators without prior ultrasound experience underwent 8 h of training on the thyroid VSI protocol and the operation of the telemedicine platform. After training, the operators scanned patients at a health center in Lima. Telediagnostic examinations were sent to the United States for remote interpretation. Standard of care thyroid ultrasound was performed by an experienced radiologist at the time of VSI examination to serve as a reference standard.

**Results:**

Novice operators scanned 121 subjects with the thyroid VSI protocol. Of these exams, 88% were rated of excellent image quality showing complete or near complete thyroid visualization. There was 98.3% agreement on thyroid nodule presence between VSI teleultrasound and standard of care ultrasound (Cohen’s kappa 0.91, *P* < 0.0001). VSI measured the thyroid size, on average, within 5 mm compared to standard of care. Readers of VSI were also able to effectively characterize thyroid nodules, and there was no significant difference in measurement of thyroid nodule size (*P* = 0.74) between VSI and standard of care.

**Conclusion:**

Thyroid VSI telediagnostic ultrasound demonstrated both excellent visualization of the thyroid gland and agreement with standard of care thyroid ultrasound for nodules and thyroid size evaluation. This system could be deployed for evaluation of palpable thyroid abnormalities, nodule follow-up, and epidemiological studies to promote global health and improve the availability of diagnostic imaging in underserved communities.

**Supplementary Information:**

The online version contains supplementary material available at 10.1007/s40618-021-01584-7.

## Introduction

Thyroid disease is ubiquitous throughout the world affecting millions of people [1]. Thyroid ultrasound is an established pillar in the diagnosis and management of palpable thyroid findings and thyroid cancer, but at least 2/3 of the world lacks access to ultrasound imaging [2–5]. While ultrasound is a cost-effective diagnostic modality, the lack of trained sonographers who require costly and time-consuming training on internal anatomy and ultrasound operation limits its deployment [6,7,8]. Furthermore, limitations in healthcare infrastructure including a lack of trained personnel and high-speed internet connections stymie many telediagnostic approaches which are otherwise viable solutions to bring imaging to underserved areas [9 –12].

**Fig. 1:**
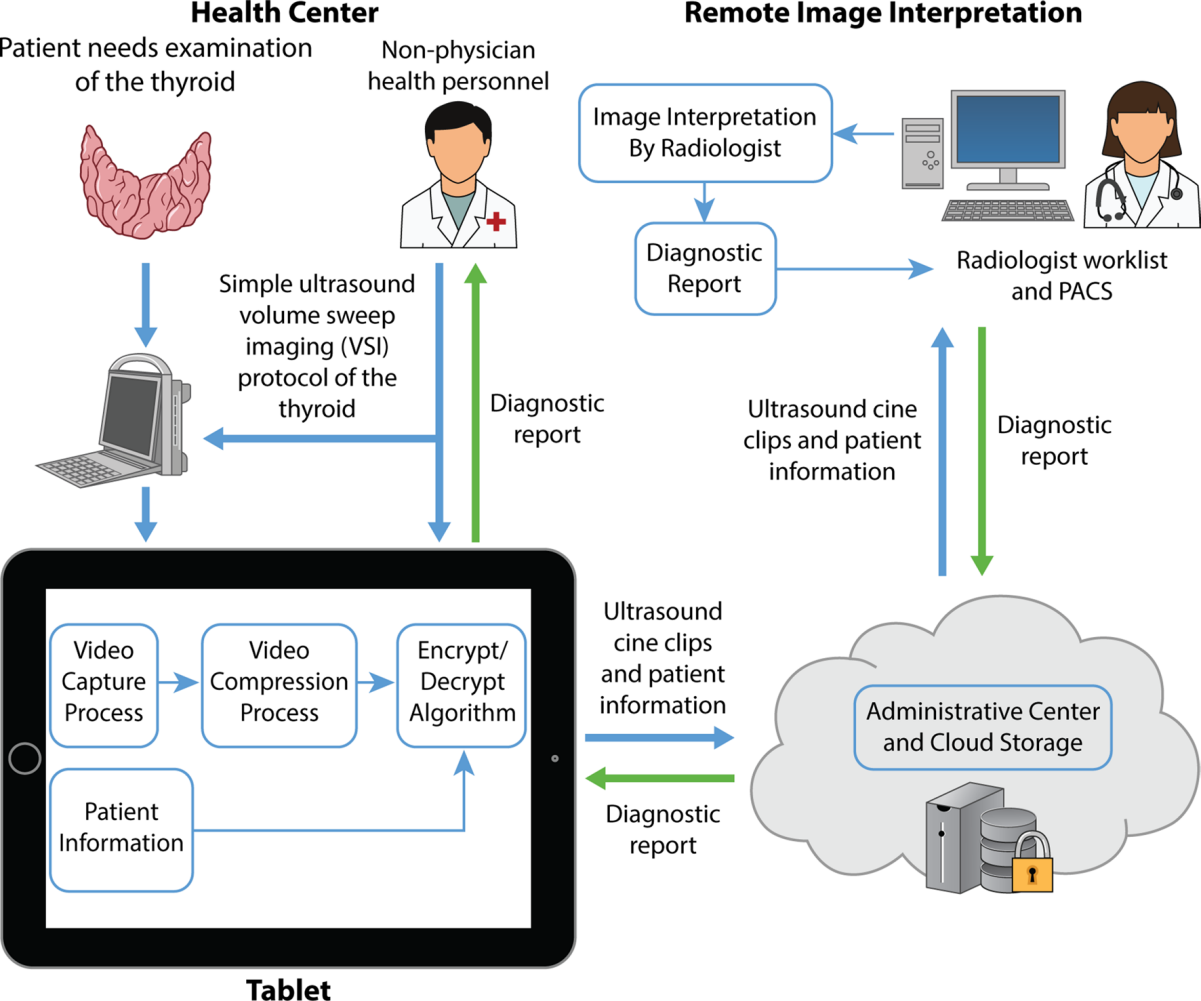
Thyroid teleultrasound. This figure shows a schematic overview of the thyroid teleultrasound system. Images acquired by individuals without prior ultrasound training are sent for remote interpretation via the use of a tablet. The imaging report is subsequently returned back to the tablet for review by the ordering clinician after interpretation by a radiologist

To address these problems, a new telediagnostic system utilizing ultrasound volume sweep imaging (VSI) has been previously piloted (Fig. 1) [13]. In this system, images are obtained by individuals without prior ultrasound experience after a few hours of training on ultrasound VSI protocols which are based on simple external body landmarks. In VSI, the operator sweeps the probe over the target area to acquire cine clips which are then sent for remote interpretation. In this asynchronous system, clips can be acquired without internet and sent over low internet bandwidths [14]. In contrast, real-time telemedicine systems require higher bandwidths for videoconferencing and the concurrent presence of a specialist which are often not available in rural and underserved areas [9, 15, 16].

Using teleultrasound to detect thyroid nodules and evaluate palpable findings could be a great asset in improving the health of the global community. Goiters remain prevalent in rural and underserved areas throughout the world; in one sample of children in northwest Ethiopia, there was a goiter prevalence of 37.6% [17–20]. If large enough, a goiter can compress the airway, and thyroid ultrasound can accurately assess thyroid size [21–23]. Goiters and palpable abnormalities also require evaluation for malignancy as studies have shown up to a 20% concurrent incidence of cancer in goiter [24, 25]. There is an increasing incidence of thyroid cancer with a global distribution including in South America where this study was conducted [26–33]. Thyroid ultrasound is a first-line examination for thyroid nodules and is used to follow and evaluate them as standard of care [34, 35]. In many areas of the world, including South America, there is a lack of information regarding thyroid nodules/cancer incidence [33, 36]. Thyroid teleultrasound could also be an important epidemiological tool to close this knowledge gap.

Our previous pilot of the teleultrasound system found that the thyroid gland was well evaluated with VSI thyroid ultrasound [13]. In the vast majority of pilot examinations, the entire thyroid gland was visualized with acceptable imaging quality. However, a relatively small sample (*n* = 22) and the lack of a ground truth for comparison were major limitations. In this study, we aimed to test the thyroid VSI protocol in a larger sample with an available ground truth reference standard. Based on the pilot data, we hypothesized that the thyroid would be completely visualized in the majority of examinations with interpretations in agreement with standard of care imaging.

## Materials and methods

### Thyroid VSI

VSI is an imaging technique designed to increase access to imaging in underserved and rural areas [37]. In VSI, the operator requires no significant anatomic knowledge or technical skill to acquire the imaging. The basic component of any VSI protocol is a sweep of the ultrasound probe over predetermined, easily recognized external body landmarks. For example, in the thyroid VSI protocol, the first sweep involves placing the probe under the chin and sweeping to the base of the neck. The cine clip of the sweep is recorded and sent for remote interpretation. In this manner, the novice operator is not responsible for any imaging interpretation. Presets of the ultrasound machine are used which further obviate the need for technical skill or adjustments of the ultrasound machine. The probe is swept at a rate of 1–2 cm/s and produces an image every 1–2 mm [13]. Additional arcing (fanning) of the probe at the beginning and at the end of each sweep maximizes visibility. When combined, all acquired cine clips comprise a full volumetric acquisition of the target organ system.

**Fig. 2:**
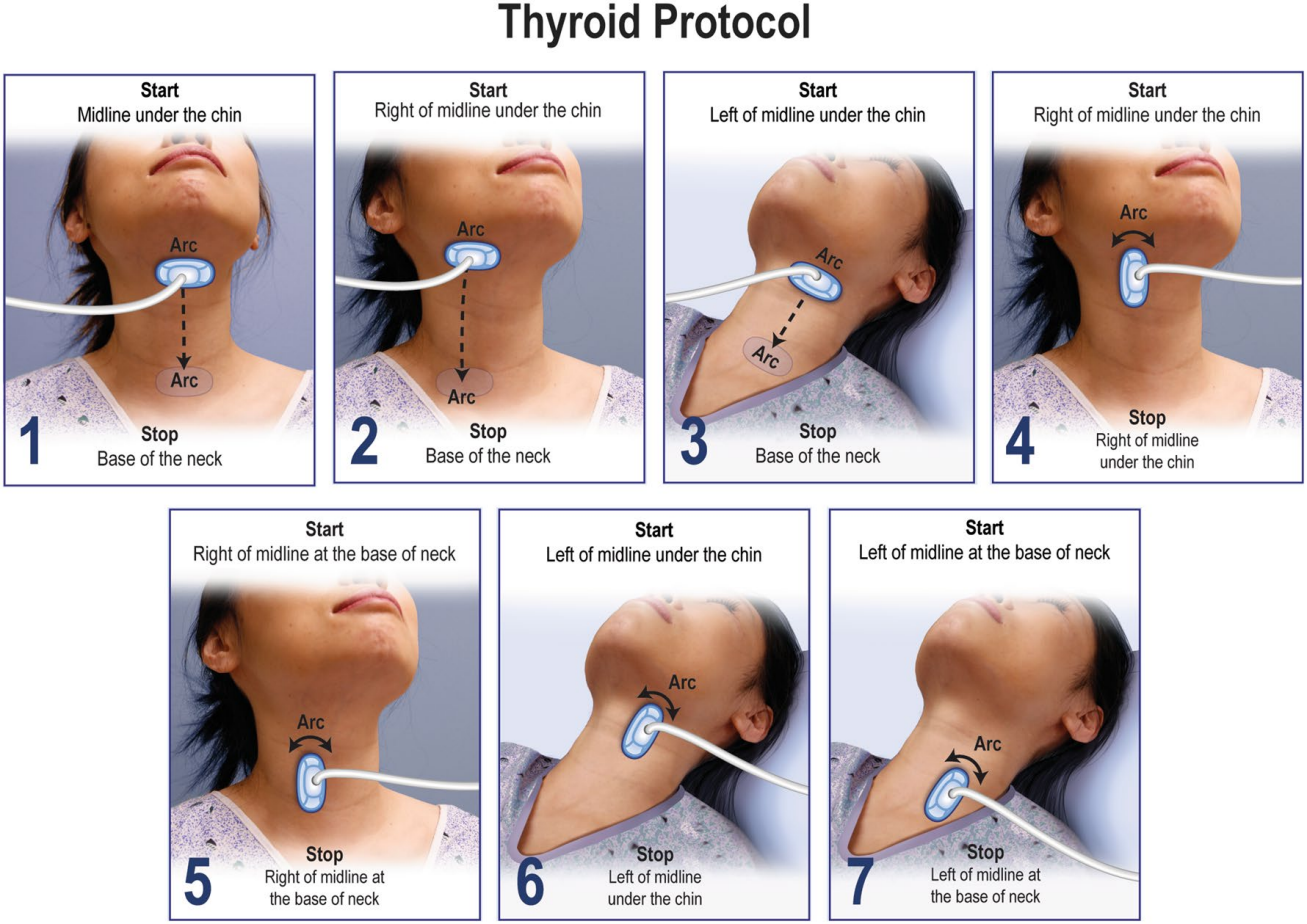
Thyroid VSI protocol. The thyroid VSI protocol involves seven steps without patient preparation. The first three steps are transverse sweeps with an arc at the start and end of each sweep to maximize visibility. The last four sweeps are longitudinal arcs in the superior and inferior right and left thyroid to provide redundancy and localization information

Thyroid, obstetric, right upper quadrant, and lung VSI protocols have all been tested with encouraging results [13, 38–40]. Training data from these studies suggest that effective VSI training is easily completed in less than 8 h. The thyroid VSI protocol can be performed within 10 min and is recommended to be performed with a high-frequency probe. There is no patient preparation required for the thyroid VSI examination which is shown in Fig. 2. Transverse sweeps of the midline, right, and left thyroid constitute the principal components of the thyroid VSI examination, while additional bilateral cine arcs of the probe are obtained in the longitudinal orientation for problem solving and redundancy. A video on how to perform the protocol and additional poster used in training are available as supplemental material (Supplemental Materials 1 and 2).

### Telemedicine system

The “Medical for Ultrasound” or MED4US system was used for this study (Medical Innovation and Technology, Peru). This system has previously been described in detail with pilot testing showing reasonable send times at low bandwidths slightly faster than dialup internet [14]. The program is installed on a Windows 10 tablet which connects to the ultrasound machine. MED4US was designed to be used by rural health workers and is user-friendly, guiding the operator into obtaining the cine clips and entering relevant patient data to be sent to the interpreting physician. The tablet encrypts, compresses, and sends the cine clips to a cloud-based storage system for remote interpretation when internet is available. Remotely, a radiologist reviews the cine clips and completes a report which is sent back to the tablet to be given to the health center and patient.

### Image acquisition

The Institutional Review Board at the Hospital Nacional Docente Madre Nino San Bartolome approved the study. Thyroid teleultrasound was piloted at the Conde de la Vega Health Center in Lima, Peru. The center serves primarily a low-income population and has a low to moderate 3G internet connection. Two ultrasound naïve trainees (a nurse and care technician) underwent didactic and hands-on training on the thyroid ultrasound protocol and telemedicine system over the course of 8 h in May 2018. Following training, both operators were certified to accurately perform the protocol by the training team. Subjects to be scanned were enrolled between June 2018 and March 2019 and were randomly recruited from patients over 18 years of age being seen at the clinic for various reasons. None of the study subjects were visiting the clinic for a primary thyroid related issue. The patient population served by the clinic is majority female.

The Mindray DP-10 ultrasound machine (Mindray, China) using standard thyroid settings with a 10 MHz linear probe was employed for both standard of care and VSI examinations. After obtaining informed consent, the attending Peruvian radiologist performed the standard of care exam in accordance with standard ultrasound consensus guidelines and completed a report [3]. Next, the thyroid ultrasound VSI exam was performed, and the VSI exam cine clip sweeps were later sent for remote interpretation in the United States. No feedback on image quality was given to the VSI operators. The radiologist who performed and interpreted the standard of care exam informed patients of any clinically significant findings.

### Remote readings

The VSI thyroid examinations were interpreted by two abdominal imaging attending radiologists with 7 and 40 years of experience, respectively. Each examination was scored on visualization of the isthmus and each thyroid lobe (less than 30% visualization, 30–50% visualization, 50–80% visualization, and greater than 80% visualization). Confidence in findings and image quality were also both scored using a three-point scale. Image quality was rated as poor, acceptable, or excellent. Excellent examinations visualized the entire or nearly entire thyroid gland with good brightness and resolution. Acceptable examinations visualized > 80% of the thyroid or had slightly limited brightness/resolution but still allowed for adequate gland or nodule assessment. Poor examinations visualized less than 80% of the total thyroid or were non-diagnostic. Confidence was scored as confident, intermediate confidence, or not confident. Both the anterior–posterior (AP) and transverse diameters of the thyroid lobes were measured. The length was not measured as the sagittal cine clips are split into upper and lower halves. Interpreters also estimated lobe size as small, normal, or large. Any nodule was measured in the largest dimension and characterized using free text with the readers commenting on the location, echogenicity, and presence of calcifications. In addition, the readers had the option of including free text comments with any image quality or study-related notes. Nodules less than 5 mm were not included in analysis as these are often clinically insignificant. After blinded readings, any discrepant cases between VSI and standard of care were examined to ascertain the source of disagreement.

### Statistical analysis

Continuous variables were summarized by mean and standard deviation, and categorical variables were summarized by rate of occurrence and 95% confidence interval (CI). Agreement between VSI and standard of care ultrasound on the presence of nodules was quantified using Cohen’s kappa. Agreement on lobe diameters was examined using intraclass correlation coefficients and Bland–Altman analysis. Intraclass correlation coefficients were calculated using a two-way random effects model for absolute agreement. Kappa values, intraclass correlation coefficients, and Bland–Altman biases were each compared to a theoretical mean of 0 using one-sample *t* tests. Nodule size in the greatest dimension was compared with a paired *t* test. All statistical analysis was performed using SPSS (v26, IBM Corporation, Armonk, NY) and MATLAB (R2019b, MathWorks, Natick, MA).

## Results

Trainees scanned 121 subjects with the thyroid VSI protocol. The average age of subjects was 33.3 ± 15 years, and 98.3% (94.2–99.8%) of subjects were female. The blinded readers stated that longitudinal sweeps were useful in 67.8% (58.7–76%) of cases for redundancy, nodule localization, and confirmation of findings. Sweeps were typically between 6 to 7 s resulting in less than one minute of cine clips for review. The raw file size for all clips combined was 6 ± 1.46 MB.

### Thyroid gland visualization and image quality

**Fig. 3:**
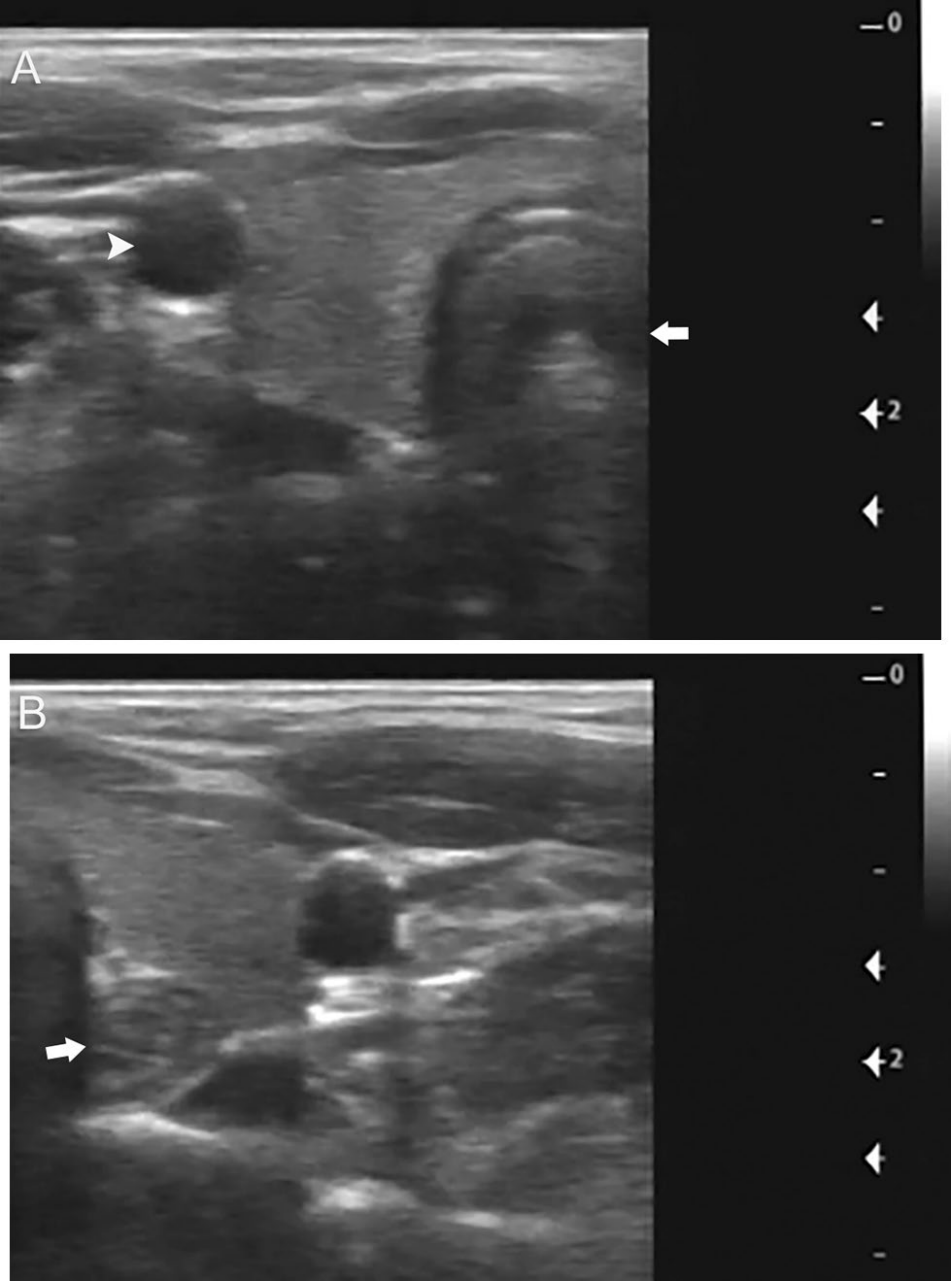
Normal thyroid on VSI. Thyroid VSI examination from an asymptomatic 65-year-old female performed by an operator after 8 h of training. **a** Normal right thyroid gland on a still image from the right transverse VSI cine clip. The trachea (arrow) and carotid artery (arrowhead) are also seen. **b** Normal left thyroid gland on a still image from the left transverse VSI cine clip. The esophagus is also seen (arrow). Supplemental Material 3 includes the cine clips from this exam. These cine clips and all other VSI examinations in this study were obtained by individuals without prior ultrasound experience

The thyroid was well visualized in all exams with the vast majority visualizing the entire gland (Table 1). The left thyroid lobe and the isthmus showed > 80% visualization in all studies. The right thyroid lobe was slightly less well visualized with 88% of the studies showing > 80% visualization. The other 12% of exams showed 50–80% visualization of the right thyroid lobe. Furthermore, readers also noted seeing the majority of the thyroid on the first single midline transverse sweep highlighting the amenability of thyroid to the volume sweep imaging approach. There was only a single examination where the first midline transverse sweep did not completely image more than 80% of the left thyroid lobe. The right thyroid lobe was less optimally evaluated on the first midline transverse sweep with 22% of the examinations showing 50–80% visualization (the other 78% showing > 80% visualization). The image quality was rated as excellent by blinded readers in 88% of cases with the thyroid well visualized with good image quality (Fig. 3 and Supplemental Material 3). Readers were confident in their estimations of lobe size and the presence of a nodule (Table 2).

**Table 1:** Image quality and visualization

Measure	Level	Percentage (95% confidence interval)
Right lobe visualized	< 30%	–
	30–50%	–
	50–80%	11.6% (6.47–18.7%)
	≥ 80%	87.6% (80.4–92.9%)
Left lobe visualized	< 30%	–
	30–50%	–
	50–80%	–
	≥ 80%	100% (97–100%)
Isthmus visualized	< 30%	–
	30–50%	–
	50–80%	–
	≥ 80%	100% (97–100%)
Image quality	Poor	–
	Acceptable	12.4% (7.11–19.6%)
	Excellent	87.6% (80.4–92.9%)

**Table 2:** Lobe characteristics and confidence

Lobe	Variable		Summary
Right	Presence of nodule		4.13% (1.36–9.38%)
	Lobe size	Small	2.48% (0.514–7.07%)
		Normal	95% (89.5–98.2%)
		Large	1.65% (0.201–5.84%)
	Confidence	Not confident	0.826% (0.0209–4.52%)
		Intermediate confidence	8.26% (4.03–14.7%)
		Confident	90.1% (83.3–94.8%)
Left	Presence of nodule		4.96% (1.84–10.5%)
	Lobe size	Small	3.31% (0.908–8.25%)
		Normal	95.9% (90.6–98.6%)
		Large	0.826% (0.0209–4.52%)
	Confidence	Not confident	0.826% (0.0209–4.52%)
		Intermediate confidence	4.13% (1.36–9.38%)
		Confident	95% (89.5–98.2%)
Isthmus	Presence of nodule		0.826% (0.0209–4.52%)
	Confidence	Not confident	–
		Intermediate confidence	3.31% (0.908–8.25%)
		Confident	96.7% (91.8–99.1%)

Values are percentage (95% confidence interval)

### Thyroid measurements and nodule agreement

**Fig. 4:**
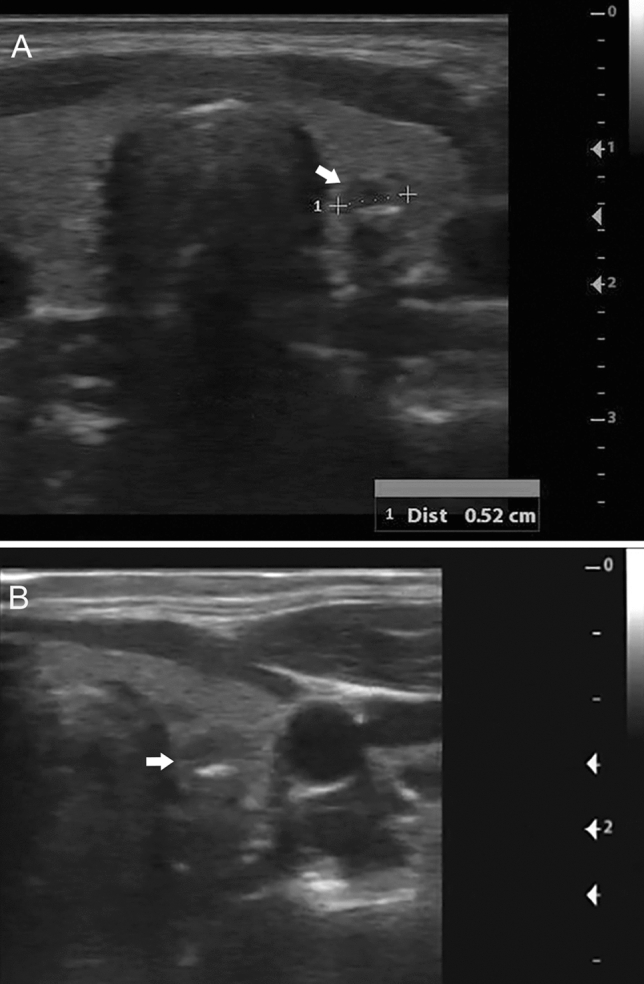
Thyroid nodule on VSI. Thyroid VSI examination from an asymptomatic 31-year-old female. **a** Standard of care image showing a 5 mm solid hypoechoic thyroid nodule (arrow) in the middle third of the left thyroid with hyperechoic calcification. **b** Left transverse VSI sweep still image showing the same solid hypoechoic nodule (arrow) also measured at 5 mm with hyperechoic calcification. The left transverse VSI cine clip has been provided as Supplemental Material 4

Thyroid size measurements on VSI were, on average, within 5 mm of standard of care imaging (Table 3). There was fair to moderate agreement with intraclass correlation coefficients varying between 0.37 and 0.58. The Bland–Altman bias varied between 2.84 and 1.07, indicating that VSI tends to result in larger measurements compared to standard of care imaging. Finally, there was excellent agreement on the presence of a nodule of 98.3% with a Cohen’s kappa of 0.91 (0.78–1, *P* < 0.0001). VSI readers were also able to confidently and effectively characterize nodule characteristics including size, location, echogenicity, and the presence of calcifications in agreement with standard of care imaging (Fig. 4 and Supplemental Material 4).

**Table 3:** Agreement on measurements

Variable	Measurement (mm)		Agreement	
	VSI	Standard of care	Intraclass correlation coefficient	Bland–Altman bias (VSI-standard of care)
Right lobe AP	16.4 ± 2.8	13.5 ± 4.36	0.37 (0.04–0.58, *p* = 0.001)	2.84 (− 5.71 to 11.4, *p* < 0.0001)
Right lobe transverse	13.1 ± 2.36	12.1 ± 2.24	0.57 (0.35–0.71, * p* < 0.0001)	1.07 (− 3.69 to 5.83, * p* < 0.0001)
Left lobe AP	14.5 ± 2.83	11.9 ± 2.97	0.42 (0.02–0.64, * p* < 0.0001)	2.54 (− 3.87 to 8.94, * p* < 0.0001)
Left lobe transverse	12.9 ± 2.32	11.2 ± 1.69	0.58 (0.01–0.79, * p* < 0.0001)	1.77 (− 1.96 to 5.5, * p* < 0.0001)
Isthmus lobe AP	3.73 ± 0.858	2.52 ± 0.965	0.48 (− 0.22 to 0.77,* p* < 0.0001)	1.21 (− 0.425 to 2.84, * p* < 0.0001)

Values are percentage (95% confidence interval). Intraclass correlation coefficients were calculated using a two-way random effects model for absolute agreement

There were 12 nodules identified on standard of care imaging—4 solid, 4 mixed cystic and solid, and 4 cystic. Of these nodules, 4 were larger than 1 cm. These studies with nodules represented 9.92% (5.23–16.7%) of exams. The average nodule size in greatest dimension was 9.8 mm ± 5.2 mm (standard deviation) on VSI and 10.1 mm ± 8 mm on standard of care imaging. There was no significant difference in nodule size (*P* = 0.74). Two nodules showed macrocalcifications which were both reported on standard of care imaging and VSI. All nodules were unknown to the patients being examined, but no follow-up information was subsequently obtained. There were only two examinations with discrepant nodule agreement between VSI and standard of care imaging. On consensus read, one examination was noted to be a missed nodule on VSI secondary to reader error. The other nodule discrepancy was not definitively resolved and may have represented a miss on standard of care imaging, but necessary standard of care images were not saved for definitive assessment. Finally, small nodules less than 5 mm were also detected on VSI and noted in the free text but not formally assessed as a part of this study. Additionally, a single goiter was identified in agreement between standard of care imaging and VSI.

## Discussion

Correcting disparities in healthcare around the world requires a multifaceted approach, and improving access to medical imaging holds much promise in this regard. Thyroid ultrasound has an important role in the evaluation of thyroid nodules and goiter which are worldwide health issues. In this study, we have shown that after only a few hours of training, individuals without prior ultrasound experience were capable of obtaining images that fully visualized the thyroid and allowed identification and characterization of nodules. Furthermore, these novice operators consistently produced diagnostic examinations without ongoing feedback over the 9-month study duration. VSI measurements of the thyroid gland were, on average, within 5 mm of standard of care measurements, showing that VSI can accurately identify thyromegaly which may require further follow-up. From an epidemiological standpoint, we identified about a 10% prevalence of a thyroid abnormality in our asymptomatic sample, including a goiter. These findings are of indeterminate pathological significance but show the potential of this approach to be useful in future epidemiological studies. Given the advent of portable ultrasound machines, it is conceivable that widespread deployment of thyroid VSI teleultrasound could be realized with the goal of improving the health of the global community. In light of our findings, we propose several uses for this system: (1) evaluating a palpable thyroid nodule or gland, (2) following a known thyroid nodule, (3) assisting in epidemiological study of thyroid disease, and (4) assisting in thyroid education and awareness (iodine deficiency and its association with goiter).

The rationale behind the first three proposed uses has already been discussed in the introduction. In terms of improving thyroid education and awareness, there are many possibilities. Thyroid literacy has been predictably noted to be lower in underserved communities [41]. As an educational tool, thyroid ultrasound may assist clinicians in teaching their patients about topics such as iodine deficiency and its relation to goiter. Having a visual connection to a health problem may improve patient compliance and understanding of health issues [42–45]. There are additional potential ancillary benefits to adopting widespread use of thyroid VSI. For example, previous studies of VSI with obstetrics showed that more patients came to clinic when ultrasound was present, resulting in higher rates of prenatal testing [46]. Similar ancillary benefits could exist for thyroid VSI as more individuals may seek care, resulting in improved health, education, and public awareness of thyroid disease.

The ultrasound novices in this study performed well acquiring examinations of universally acceptable to excellent image quality. While we did not notice a decrease in image quality over the study period, the use of refresher courses on VSI could also be considered as a way to ensure continued quality. Even with excellent image quality in the majority of examinations, it is possible some small nodules may be better served by dedicated sonographic evaluation with high-resolution imaging by an expert. Similarly, increased body habitus or otherwise abnormal neck anatomy may limit the VSI protocol’s ability to assess the thyroid. Future studies could assess the protocol’s performance in these circumstances. In addition, the number of nodules larger than 1 cm in this sample was relatively small. Future studies might be conducted in thyroid clinics with a sample of more disease or in populations with goiter. Length measurements of the thyroid are currently not obtainable due to the split nature of the longitudinal clips. This could be potentially solved with advanced imaging processing or revised future iterations of the protocol. Artificial intelligence could also allow for identification of nodules and thyroid size at the time of study.

Although there are characteristic features of hyperthyroidism and hypothyroidism on ultrasound, imaging is not recommended in the routine evaluation of these conditions [46,48, 49]. As with standard of care thyroid ultrasound, there exists the theoretical risk for over-screening of thyroid nodules [50–52]. This issue may be mitigated by the increasing use of the Thyroid Imaging Reporting and Data System (TI-RADS) which improves accuracy and may reduce the number of biopsies prompted by ultrasound [53]. VSI can adequately assess for the features of TI-RADS allowing for standardized management of findings. Evidence also suggests that it is important to note the clinical context of each individual patient when considering biopsy [54]. Our telediagnostic system also provides this relevant clinical information to the interpreting provider. At this time, we are not advocating this protocol be used to screen asymptomatic people outside of epidemiological studies. Some clinicians may be concerned regarding legal exposure from use of VSI examinations. In general, the alternative to clinical use of thyroid VSI in many rural and underserved areas would be no imaging at all. Given the potential clinical benefits in this context, significant legal exposure is not anticipated. Furthermore, review of the legal literature shows no lawsuits related to point of care ultrasound use, and, in fact, lawsuits were identified when ultrasound was failed to be performed [55, 56].

Thyroid disease is a prevalent health issue worldwide with a majority of the world lacking access to diagnostic imaging for its evaluation [2]. The examinations in this study obtained by individuals without prior ultrasound experience showed excellent agreement with standard of care imaging for thyroid size and nodule evaluation. As rates of thyroid cancer continue to increase, thyroid VSI telediagnostic ultrasound could be a cost-effective asset both in determining clinical management and in conducting epidemiological study. Deployment of this system is feasible with significant potential to improve medical care of those in need, especially in rural and underserved areas.

## Supplementary Information

Below is the link to the electronic supplementary material.

Supplemental Material 1. Thyroid training video. Training video on the thyroid VSI protocol used in this study produced by Medical Imaging Ministries of the Americas and Medical Innovation and Technology (MOV 52604 KB)

Supplemental Material 2. Thyroid training poster. Poster used in the original thyroid training session for this study produced by Medical Imaging Ministries of the Americas and Medical Innovation and Technology (TIF 3195 KB)

Supplemental Material 3. Normal thyroid VSI examination. Sweeps from the same asymptomatic female as seen in Figure 3 constituting a full representative volumetric examination of the thyroid. Sweeps are listed in order as demonstrated in Figure 2. The first three sweeps are transversely oriented and the last four are longitudinally oriented (MP4 1136 KB)

Supplementary file4 (MP4 956 KB)

Supplementary file5 (MP4 1307 KB)

Supplementary file6 (MP4 571 KB)

Supplementary file7 (MP4 600 KB)

Supplementary file8 (MP4 690 KB)

Supplementary file9 (MP4 802 KB)

Supplemental Material 4. Thyroid nodule on VSI. Transverse sweep through the left thyroid demonstrating the same hypoechoic solid nodule in the middle third of the left thyroid with macrocalcification (MP4 725 KB)

## Data Availability

The data sets generated in this study are not publically available as an additional measure to protect subject privacy. However, all relevant data have been presented in this article. Please address any inquiries regarding data use to Dr. Castaneda.

## References

[1] The Lancet D, Endocrinology (2013). The untapped potential of the thyroid axis. Lancet Diabetes Endocrinol.

[2] World Radiography Day: Two-Thirds of the World's Population has no Access to Diagnostic Imaging. Pan American Health Organization. https://www.paho.org/hq/index.php?option=com_content&view=article&id=7410:2012-dia-radiografia-dos-tercios-poblacion-mundial-no-tiene-acceso-diagnostico-imagen&Itemid=1926&lang=en#:~:text=%2D%20The%20use%20of%20X%2Drays,no%20access%20to%20diagnostic%20imaging.&text=It%20is%20used%20for%20diagnostic%2C%20preventive%2C%20and%20therapeutic%20purposes. Accessed 30 Oct 2020

[3] (2013)AIUM practice guideline for the performance of a thyroid and parathyroid ultrasound examination. J Ultrasound Med 32(7):1319–1329. 10.7863/ultra.32.7.1319 10.7863/ultra.32.7.1319 23804357

[4] Chaudhary V, Bano S (2013). Thyroid ultrasound. Indian J Endocrinol Metab.

[5] Maru DS, Schwarz R, Jason A, Basu S, Sharma A, Moore C (2010). Turning a blind eye: the mobilization of radiology services in resource-poor regions. Glob Health.

[6] Mollura DJ, Lungren MP (2014). Radiology in global health: strategies, implementation, and applications.

[7] Mollura DJ, Mazal J, Everton KL, Group R-ACW (2013) White paper report of the 2012 RAD-AID conference on International Radiology for Developing Countries: planning the implementation of global radiology. J Am Coll Radiol 10(8):618–624. 10.1016/j.jacr.2013.01.019 10.1016/j.jacr.2013.01.019 23583085

[8] Ngoya PS, Muhogora WE, Pitcher RD (2016). Defining the diagnostic divide: an analysis of registered radiological equipment resources in a low-income African country. Pan Afr Med J.

[9] Britton N, Miller MA, Safadi S, Siegel A, Levine AR, McCurdy MT (2019). Tele-ultrasound in resource-limited settings: a systematic review. Front Public Health.

[10] Kim T, Zuckerman JE (2019). Realizing the potential of telemedicine in global health. J Glob Health.

[11] Popov V, Popov D, Kacar I, Harris RD (2007). The feasibility of real-time transmission of sonographic images from a remote location over low-bandwidth Internet links: a pilot study. AJR Am J Roentgenol.

[12] Scott Kruse C, Karem P, Shifflett K, Vegi L, Ravi K, Brooks M (2018). Evaluating barriers to adopting telemedicine worldwide: a systematic review. J Telemed Telecare.

[13] Marini TJ, Oppenheimer DC, Baran TM, Rubens DJ, Toscano M, Drennan K, Garra B, Miele FR, Garra G, Noone SJ, Tamayo L, Carlotto C, Trujillo L, Waks E, Garra K, Egoavil MS, Berrospi J, Castaneda B (2020). New ultrasound telediagnostic system for low-resource areas: pilot results from Peru. J Ultrasound Med.

[14] Ferrer J, Chaumont T, Trujillo L, Fernandez I, Guerrero J, Stewart P, Garra G, Campos MF, Garra K, Stephens N, Harley C, Jacobo S, Waks E, Miele F, Garra B, Castaneda B (2017). New tele-diagnostic model using volume sweep imaging for rural areas. Conf Proc IEEE Eng Med Biol Soc.

[15] Health Care Broadband in America (2010) Federal Communications Commission. https://www.fcc.gov/document/obi-technical-paper-no-5-health-care-broadband-america

[16] Measuring Digital Development Facts and Figures (2019) International Telecommunication Union. https://www.itu.int/myitu/-/media/Publications/2020-Publications/Measuring-digital-development-2019.pdf

[17] Mesele M, Degu G, Gebrehiwot H (2014). Prevalence and associated factors of goiter among rural children aged 6–12 years old in Northwest Ethiopia, cross-sectional study. BMC Public Health.

[18] Pretell EA, Moncloa F, Salinas R, Kawano A, Guerra-Garcia R, Gutierrez L, Beteta L, Pretell J, Wan M (1969). Prophylaxis and treatment of endemic goiter in Peru with iodized oil. J Clin Endocrinol Metab.

[19] Stanbury JB, Kevany JP (1970). Iodine and thyroid disease in Latin America. Environ Res.

[20] Unnikrishnan AG, Menon UV (2011). Thyroid disorders in India: an epidemiological perspective. Indian J Endocrinol Metab.

[21] Abraham D, Singh N, Lang B, Chan WF, Lo CY (2007). Benign nodular goitre presenting as acute airway obstruction. ANZ J Surg.

[22] Başoğlu M, Öztürk G, Aydınlı B, Yıldırgan M, Atamanalp SS, Celebi F (2009). Benign nodular goiter causing upper airway obstruction. Eurasian J Med.

[23] Sajid B, Rekha K (2017). Airway management in patients with tracheal compression undergoing thyroidectomy: a retrospective analysis. Anesth Essays Res.

[24] Pang HN, Chen CM (2007). Incidence of cancer in nodular goitres. Ann Acad Med Singap.

[25] Smith JJ, Chen X, Schneider DF, Broome JT, Sippel RS, Chen H, Solórzano CC (2013). Cancer after thyroidectomy: a multi-institutional experience with 1,523 patients. J Am Coll Surgeons.

[26] Borges AKdM, Miranda-Filho A, Koifman S, Koifman RJ (2017). Thyroid cancer incidences from selected south america population-based cancer registries: an age-period-cohort study. J Glob Oncol.

[27] Deng Y, Li H, Wang M, Li N, Tian T, Wu Y, Xu P, Yang S, Zhai Z, Zhou L, Hao Q, Song D, Jin T, Lyu J, Dai Z (2020). Global burden of thyroid cancer from 1990 to 2017. JAMA Netw Open.

[28] Kitahara CM, Sosa JA (2016). The changing incidence of thyroid cancer. Nat Rev Endocrinol.

[29] López Gavilanez E, Bautista Litardo N, Navarro Chávez M, Hernández Bonilla M, Segale Bajaña A (2020). Thyroid cancer in Ecuador. BMC Cancer.

[30] Pellegriti G, Frasca F, Regalbuto C, Squatrito S, Vigneri R (2013). Worldwide increasing incidence of thyroid cancer: update on epidemiology and risk factors. J Cancer Epidemiol.

[31] Salazar-Vega J, Ortiz-Prado E, Solis-Pazmino P, Gómez-Barreno L, Simbaña-Rivera K, Henriquez-Trujillo AR, Brito JP, Toulkeridis T, Coral-Almeida M (2019). Thyroid cancer in Ecuador, a 16 years population-based analysis (2001–2016). BMC Cancer.

[32] Seib CD, Sosa JA (2019). Evolving understanding of the epidemiology of thyroid cancer. Endocrinol Metab Clin N Am.

[33] Sierra MS, Soerjomataram I, Forman D (2016). Thyroid cancer burden in Central and South America. Cancer Epidemiol.

[34] Marqusee E, Benson CB, Frates MC, Doubilet PM, Larsen PR, Cibas ES, Mandel SJ (2000). Usefulness of ultrasonography in the management of nodular thyroid disease. Ann Intern Med.

[35] Tollin SR, Mery GM, Jelveh N, Fallon EF, Mikhail M, Blumenfeld W, Perlmutter S (2000). The use of fine-needle aspiration biopsy under ultrasound guidance to assess the risk of malignancy in patients with a multinodular goiter. Thyroid.

[36] Salazar-Vega J, Ortiz-Prado E, Solis-Pazmino P, Gomez-Barreno L, Simbana-Rivera K, Henriquez-Trujillo AR, Brito JP, Toulkeridis T, Coral-Almeida M (2019). Thyroid cancer in Ecuador, a 16 years population-based analysis (2001–2016). BMC Cancer.

[37] DeStigter K, Morey G, Garra B, Rielly M, Anderson M, Kawooya MG, Matovu A, Miele F Low-cost teleradiology for rural ultrasound. In: Proceedings of the IEEE global humanitarian technology conference (GHTC). Seattle, Washington, Oct 30–Nov 1, 2011. IEEE Press, pp 290–295

[38] Toscano M, Marini TJ, Drennan K et al (2021) Testing telediagnostic obstetric ultrasound in Peru: a new horizon in expanding access to prenatal ultrasound. BMC Pregnancy Childbirth 21:328 (2021). 10.1186/s12884-021-03720-w 10.1186/s12884-021-03720-w PMC8074497 33902496

[39] Dougherty A, Kasten M, DeSarno M, Badger G, Streeter M, Jones DC, Sussman B, DeStigter K (2020). Validation of a telemedicine quality assurance method for point-of-care obstetric ultrasound used in low-resource settings. J Ultrasound Med.

[40] Marini​ T, Castaneda B, Baran T, O’Connor T, Garra B, Tamayo L, Zambrano M, Carlotto C, Trujillo L, Kaproth-Joslin K (2019) Lung ultrasound volume sweep imaging for pneumonia detection in rural areas: piloting training in rural Peru. J Clin Imaging Sci 9(35). https://clinicalimagingscience.org/lung-ultrasound-volume-sweep-imaging-for-pneumonia-detection-in-rural-areas-piloting-training-in-rural-peru/10.25259/JCIS_29_2019 PMC6737249 31538033

[41] Perumal SS, Prasad S, Surapaneni KM, Joshi A (2015). Health information-seeking behavior among hypothyroid patients at Saveetha Medical College and Hospital. Ethiop J Health Sci.

[42] Alamantariotou K, Zisi D (2010). Consumer health informatics and interactive visual learning tools for health. Int J Electron Healthc.

[43] Beacom AM, Newman SJ (2010). Communicating health information to disadvantaged populations. Fam Community Health.

[44] Entwistle V, Williams B (2008). Health literacy: the need to consider images as well as words. Health Expect Int J Public Particip Health Care Health Policy.

[45] Peregrin T (2010). Picture this: visual cues enhance health education messages for people with low literacy skills. J Am Diet Assoc.

[46] Ross AB, DeStigter KK, Rielly M, Souza S, Morey GE, Nelson M, Silfen EZ, Garra B, Matovu A, Kawooya MG (2013). A low-cost ultrasound program leads to increased antenatal clinic visits and attended deliveries at a health care clinic in rural Uganda. PLoS ONE.

[47] Ceylan I, Yener S, Bayraktar F, Secil M (2014). Roles of ultrasound and power Doppler ultrasound for diagnosis of Hashimoto thyroiditis in anti-thyroid marker-positive euthyroid subjects. Quant Imaging Med Surg.

[48] Pedersen OM, Aardal NP, Larssen TB, Varhaug JE, Myking O, Vik-Mo H (2000). The value of ultrasonography in predicting autoimmune thyroid disease. Thyroid.

[49] Trimboli P, Rossi F, Condorelli E, Laurenti O, Ventura C, Nigri G, Romanelli F, Guarino M, Valabrega S (2010). Does normal thyroid gland by ultrasonography match with normal serum thyroid hormones and negative thyroid antibodies?. Exp Clin Endocrinol Diabetes.

[50] Morris LG, Sikora AG, Tosteson TD, Davies L (2013). The increasing incidence of thyroid cancer: the influence of access to care. Thyroid.

[51] Sanabria A, Kowalski LP, Shah JP, Nixon IJ, Angelos P, Williams MD, Rinaldo A, Ferlito A (2018). Growing incidence of thyroid carcinoma in recent years: factors underlying overdiagnosis. Head Neck.

[52] Vigneri R, Malandrino P, Vigneri P (2015). The changing epidemiology of thyroid cancer: why is incidence increasing?. Curr Opin Oncol.

[53] Grant EG, Tessler FN, Hoang JK, Langer JE, Beland MD, Berland LL, Cronan JJ, Desser TS, Frates MC, Hamper UM, Middleton WD, Reading CC, Scoutt LM, Stavros AT, Teefey SA (2015). Thyroid ultrasound reporting lexicon: white paper of the ACR Thyroid Imaging, Reporting and Data System (TIRADS) Committee. J Am Coll Radiol.

[54] Magri F, Chytiris S, Croce L, Molteni M, Bendotti G, Gruosso G, Tata Ngnitejeu S, Agozzino M, Rotondi M, Chiovato L (2020). Performance of the ACR TI-RADS and EU TI-RADS scoring systems in the diagnostic work-up of thyroid nodules in a real-life series using histology as reference standard. Eur J Endocrinol.

[55] Blaivas M, Pawl R (2012). Analysis of lawsuits filed against emergency physicians for point-of-care emergency ultrasound examination performance and interpretation over a 20-year period. Am J Emerg Med.

[56] Stolz L, O'Brien KM, Miller ML, Winters-Brown ND, Blaivas M, Adhikari S (2015). A review of lawsuits related to point-of-care emergency ultrasound applications. West J Emerg Med.

