# Supplementary figures and images for: Testing telediagnostic thyroid ultrasound in Peru: a new horizon in expanding access to imaging in rural and underserved areas
Source: J Endocrinol Invest. 2021 May 10;44(12):2699–708. doi: 10.1007/s40618-021-01584-7 (PMC8572222; doi:10.1007/s40618-021-01584-7)

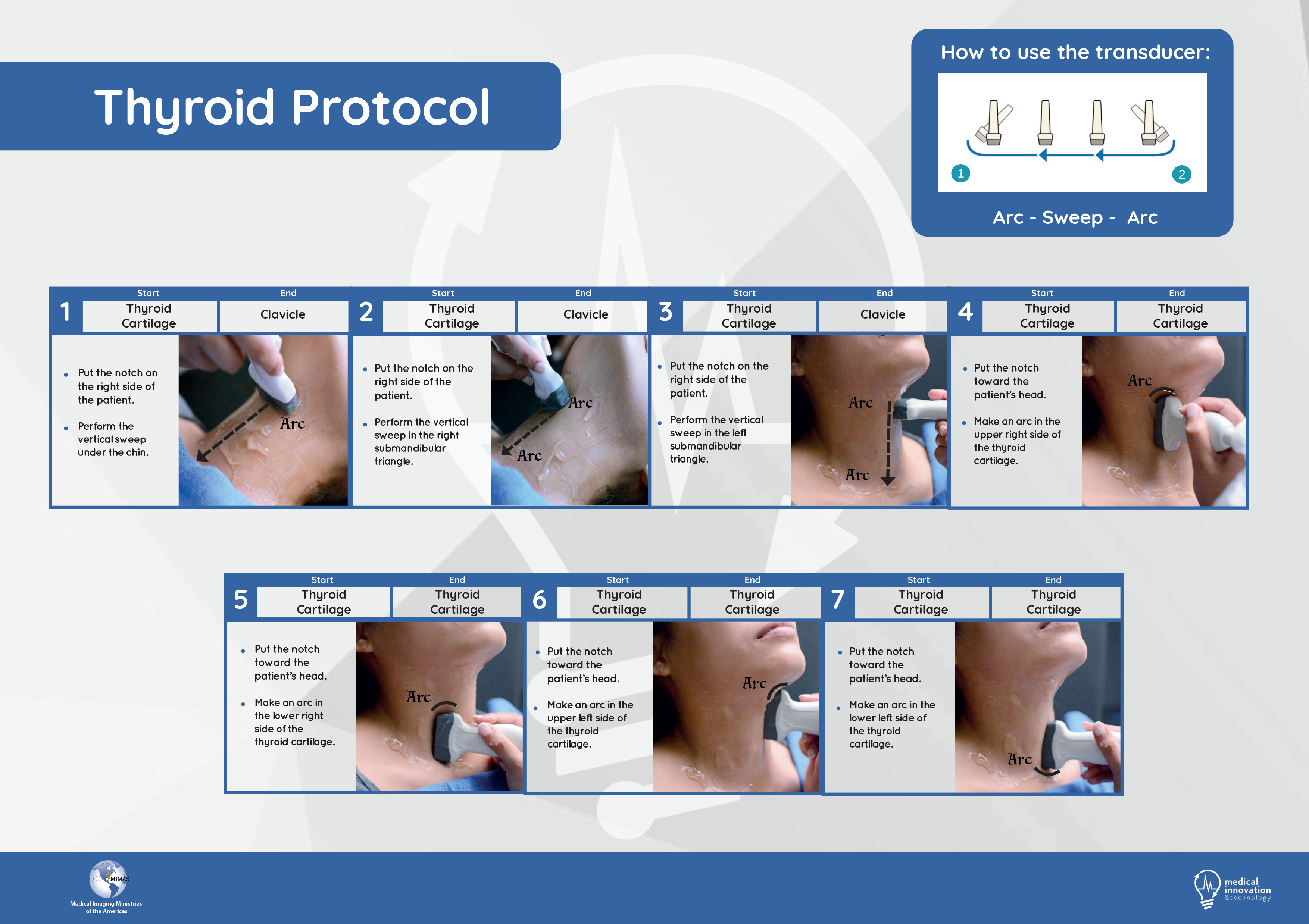

Supplement: Supplementary file 2 — Supplemental Material 2. Thyroid training poster. Poster used in the original thyroid training session for this study produced by Medical Imaging Ministries of the Americas and Medical Innovation and Technology (TIF 3195 KB) [file 40618_2021_1584_MOESM2_ESM.tif]
